# Effect of a nutraceutical combination on sleep quality among people with impaired sleep: a randomised, placebo-controlled trial

**DOI:** 10.1038/s41598-024-58661-z

**Published:** 2024-04-05

**Authors:** Sebastián Antonio Gutiérrez-Romero, Erika Sofía Torres-Narváez, Adrián Camilo Zamora-Gómez, Silvana Castillo-Castillo, Angela Liliana Latorre-Velásquez, Carolina Betancourt-Villamizar, Carlos O. Mendivil

**Affiliations:** 1 School of Medicine, Universidad de los Andes, Carrera 7 No 116-05, Of 413, 110111 Bogotá, Colombia; 2 Department of Neurology, Hospital Universitario Fundación Santa Fe de Bogotá, Bogotá, Colombia; 3 Research and Development, Team Foods Colombia, Bogotá, Colombia; 4 Section of Endocrinology, Department of Internal Medicine, Hospital Universitario Fundación Santa Fe de Bogotá, Bogotá, Colombia

**Keywords:** Sleep disorders, Translational research, Risk factors

## Abstract

In this randomised, placebo-controlled trial, adults with impaired sleep (Pittsburgh Sleep Quality Index ≥ 5) were randomly assigned using a minimization algorithm to receive a formulation containing l-theanine plus lemon balm, valerian, and saffron extracts, or placebo, during 6 weeks. Objective sleep quality parameters were measured using an actigraphy device. We enrolled and randomised 64 individuals, 31 from the active group and 27 from the placebo group completed the 6 week follow-up. Mean sleep efficiency remained unmodified in the active group, and increased by 3% in the placebo group, the between-group difference in the change was not statistically significant (p = 0.49). Total sleep time also improved more with placebo (13.0 vs. 1.33 min, p = 0.66). Time wake after sleep onset (WASO) decreased more in the active group (4.6% vs. 2.4%), but the difference was not significant (p = 0.33). Mean PSQI decreased by 3.11 points (32.3%) in the active group, and by 3.86 points (39.5%) in the placebo group (p = 0.41). SF-36 increased more with placebo (+ 18.3 in active, + 32.1 in placebo, p = 0.68). Salivary cortisol remained unchanged in both groups. No serious adverse events were reported. Among adults with impaired sleep, a nutraceutical combination did not improve objective or subjective sleep parameters more than a placebo infusion.

## Introduction

Sleep is a fundamental biological process for human health^1^. During sleep, the human organism saves energy, repairs tissues, restores energy sources and develops metabolic and immunologic adaptations^2^. Sleep also plays a fundamental role in memory consolidation^3^ and in the clearance of beta-amyloid and other noxious metabolites^4^.

From an evolutionary perspective, sleep shows substantial variation among species, as it is determined largely by environmental conditions and feeding requirements^5^. In human beings, many additional elements may influence sleep, including social, environmental, and individual factors^6^. At the societal level, public policies, exposure to technology, and the 24/7 society characteristic of a globalized labour market, condition sleep habits. At the environmental level, factors like living conditions, work schedules, neighbourhood safety and prevailing cultural norms also impinge on sleep. Finally, the genetic makeup, beliefs, attitudes, and behaviours of each individual are key determinants of their sleep patterns^6^. All these features make sleep a complex and labile experience for humans.

Data from the 2021 Philips Global Sleep Survey show that sleep disturbances are an underdiagnosed and poorly controlled problem worldwide. Only 55% of adults were completely or somewhat satisfied with their sleep^7^. The majority of adults sleep on average 1.2 h less than recommended (6.8 h vs. 7–9 h), and eight out of ten want to improve their quality of sleep, but less than half of them (40%) have consulted a health professional for this. Data from an urban population in Colombia showed that almost 60% of adults had a sleep concern, of whom 45% might require medical management^8^.

Given the multifactorial nature of sleep, and the fact that sleep disorders can be successfully managed when properly diagnosed^9^, multiple interventions have been tested to improve sleep quality. One such group of interventions is nutraceutical products, supplements derived from plants or plant extracts containing biological components that potentially improve different aspects of sleep quality^10^. The public interest and exposure to advertising of nutraceutical products is growing rapidly^11^, but there is a paucity of data from quality randomised trials assessing the efficacy and safety of nutraceutical products and their combinations among people with sleep problems^12,13^. This scarcity of high-quality data on the combined efficacy of sleep supplements, and their large and increasing popularity, motivated us to undertake of this study.

l-theanine is an organic compound from tea, whose effects on sleep have been analyzed extensively. Different studies have found positive effects of l-theanine on relaxation, sleep quality, cognitive performance, and mood^14^. Lemon balm (*Melissa officinalis*) extract is postulated to increase bioavailability of the inhibitory neurotransmitter GABA, by reducing the activity of its degrading enzyme^15^, potentially improving sleep quality among patients with insomnia and anxiety^16^. Meanwhile, valerian (*Valeriana officinalis*), has been tested as a sleep aid in over 40 interventional studies, included in a recent meta-analysis^17^ that found significant positive effects on sleep. Lastly, positive effects of saffron (*Crocus sativus*) on sleep have also been reported, with a meta-analysis of eight clinical trials in adults reporting improved sleep quality and minimal adverse effects^18^.

On this basis, we aimed to assess the impact of a nutraceutical formulation that combines extracts from plants for which positive impacts on sleep have been reported (green tea, lemon balm, valerian and saffron)^14–18^, among adults with impaired sleep. The novelty of the study resides in that it formally tested the efficacy and safety of a combination of herbal supplements for sleep improvement, which are extensively used worldwide.

## Methods

### Study design

This was a randomised, double-blind, placebo controlled clinical trial conducted between December 2022 and June 2023 in Bogota, Colombia. The purpose of the study was to assess the efficacy and safety of a nutraceutical formulation aimed at improving sleep quality, among people with impaired sleep (see inclusion criteria). The study protocol was approved by the IRB Riesgo de Fractura S.A.*,* according to minute CEI-51 of December 15, 2022. The study was registered at https://www.clinicaltrials.gov under number NCT05609890 on 08/11/2022. All participants underwent an informed consent procedure and provided written informed consent before participation. All study procedures were executed in compliance with regulations for health research dictated by resolution 8430-1993 of the Colombian Ministry of Health and with the principles stated by the Helsinki Declaration of 1964 and its later amendments.

### Participants

Participants were men and women aged 18 or older with impaired sleep (defined as a Pittsburgh sleep quality score [PSQI] ≥ 5), not receiving any specific treatment for sleep improvement at the time of enrolment and not planning to take any for the next 2 months. The exclusion criteria were: history of a specific sleep disorder according to the diagnostic and statistical manual of mental disorders—fifth edition (DSM-5), history of an anxiety disorder, depression or other psychiatric or neurological condition, uncontrolled hypothyroidism [assessed by a measurement of basal thyroid-stimulating hormone (TSH)], history of cortisol deficit or excess, alcohol intake greater than two standard drinks (30 g of alcohol) per day, caffeine intake greater than 400 mg per day (3 cups of coffee or 1 energy drink per day), frequent sleep deprivation (shift work or need to skip sleep) over the last 2 months, smoking or use of tobacco products, use of recreational drugs, and childbearing desire among women. We did not set an upper PSQI cutoff as an exclusion criterion.

### Randomisation and masking

Eligible individuals who manifested their wish to take part in the study were contacted for screening by phone, filled a PSQI questionnaire to verify eligibility and provided their informed consent. Then, they underwent a 1 week run-in phase, in which all received the active nutraceutical formulation. Those with adherence ≥ 85% went on to a washout week, during which they wore a Fitbit Charge 5 actigraphy device, linked to a cloud-based server for collection of sleep variables, and kept a detailed sleep diary. Participants who completed the run-in and washout phases attended an in-person visit in which baseline vital signs, anthropometric variables and blood and saliva samples were collected. Weight was measured using a Tanita innerscan body composition monitor, and height was measured with a Seca portable stadiometer. Body-mass index (BMI) was calculated as the ratio of weight in kilograms to the square of the height in meters. Blood pressure was measured in the right arm after fifteen minutes of seating using a Welch Allyn DuraShock DS45 sphygmomanometer. Heart rate was recorded using a fully charged pulse oximeter. All measurements were conducted by trained staff members. Blood samples were used to analyse plasma glucose levels (biosystems cat 11803), HbA1c (NycoCard cat 1116083), lipid profile (biosystems total cholesterol cat11805, biosystems triglycerides cat 11828, biosystems HDL cholesterol cat 11523), liver enzymes (biosystems alanine aminotransferase cat 11832, biosystems aspartate aminotransferase cat 11830), creatinine (biosystems cat 11802), urea (biosystems cat 11536), bilirubin (biosystems cat 11515) and thyroid-stimulating hormone (by a fluorescent immunoassay in an Abbott ARCHITECT reader). Saliva samples were collected to measure cortisol (by an electrochemiluminescent assay in an Abbott ARCHITECT reader).

In the same visit, participants fulfilled a short form—36 questions (SF-36) quality of life questionnaire and were randomised using a minimization algorithm to the active intervention or placebo group with minimization by age (< 40 or ≥ 40), sex, and basal PSQI (< 8 or ≥ 8). Minimization is a randomization technique in which each new enrolled participant is assigned to one intervention group in a way that minimizes the overall differences between groups in a set of variables (in our case, age, sex, and basal PQI)^19^. Participants, data collectors and researchers who performed statistical analysis were unaware of the group allocation codes. The nutraceutical formulation and placebo were both powdered and contained in sealed opaque envelopes. Even though the appearance and taste of the two interventions were not identical, participants and researchers who provided the envelopes and assessed the results were unaware of which colour or taste corresponded to which intervention.

### Procedures

The nutraceutical formulation was a powder mixture of green tea (*Camellia sinensis*) extract, equivalent to 200 mg of l-theanine, lemon balm (*Melissa officinalis*) leaf extract (400 mg), valerian (*Valeriana officinalis*) extract (500 mg), saffron extract (Lepticrosalides^®^) (14 mg), and excipients. The placebo group received a powder mixture of the excipients that was contained lemongrass, peppermint, and mint flavourings, but not their active components.

**Figure 1:**
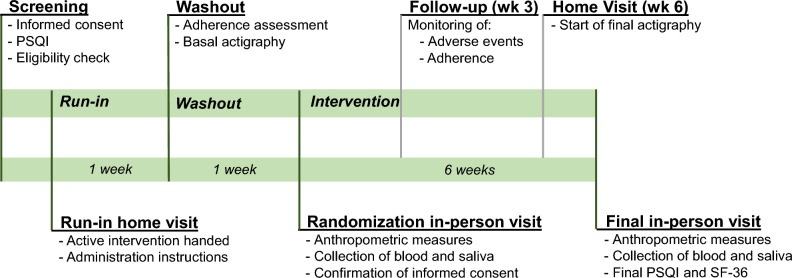
Study timeline.

Participants were instructed to pour the full content of each envelope into 200 ml of warm water and drink it 1 h before going to bed. They were also advised not to add anything else to this beverage, and to store all empty envelopes in order to return them to the study staff for assessment of adherence. In the randomization visit, each participant received enough envelopes of their allocated intervention for the study duration (6 weeks). Participants received a diary book to keep track of their taken doses of the intervention and were asked to record any new symptom in this book, and to communicate it immediately to the study staff. All participants received a phone call at week 3 of the intervention phase, in which they were inquired about adverse events and adherence. At the end of week 5 for each participant, we delivered the actigraphy device at their home in order to collect the final values of actigraphy measures during week 6. At the end of week 6, participants attended a final in-person visit in which they returned the unused intervention doses (which were used to calculate adherence), returned the actigraphy device, fulfilled again the PSQI and SF-36 questionnaires, and provided final blood and saliva samples. Figure 1 summarizes the study timeline.

### Outcomes

Our primary outcome was the between-group difference in the change in sleep efficiency (final-basal) over 6 weeks. Sleep efficiency is defined as the ratio of total time asleep to total time in bed (expressed as a percentage). Total time in bed was calculated by subtracting the go-to-sleep time from the time when the participant was no longer trying to sleep (rise time), both reported in the sleep diary. Patients were instructed to be as accurate as possible in registering the exact time they attempted to fall asleep, and the exact time they woke up and no longer intended to sleep^20^. Total time asleep was extracted from actigraphy data registered by the Fitbit Charge 5. We selected the Fitbit Charge 5, as this actigraphy device has shown comparable recordings of sleep–wake states and sleep stage composition relative to the gold standard (polysomnography)^21,22^.

One secondary outcome was the group difference in change in PSQI, collected from participants at the screening contact and at the final visit. The PSQI^23,24^ is an 18-item, ten-section instrument that assesses seven domains related to sleep quality, namely subjective sleep quality, sleep latency, sleep duration, habitual sleep efficiency, sleep disturbances, use of sleep medications, and daytime dysfunction; all of them over the last month. PSQI scores range from 0 to 21, with higher scores indicating worse sleep quality. Another secondary outcome was the group difference in change in wake after sleep onset (WASO), defined as the total number of minutes that the participant was awake after having initially fallen asleep^25^. WASO was obtained directly from actigraphy data. We also analysed as secondary outcomes the group difference in salivary cortisol (measured at the exact same time of the day for each participant), and the change in the 36-item short form survey score (SF-36). SF-36^26,27^ is a questionnaire that assesses health-related quality of life reflected in eight domains: physical functioning, physical role, bodily pain, general health, vitality, social functioning, emotional role, and mental health^28^. Higher SF-36 scores represent better health and functioning. Safety was monitored by inquiring in detail about adverse events in all study visits, and by assessing changes in liver and kidney function tests. All measurements were undertaken in a centralized laboratory.

### Statistical analyses

Sample size was calculated using the expression for two-arm trials with a continuous outcome^29^. For an assumed variability in sleep efficiency of 2.3%^30^, a sample size of 60 participants would provide us with 95% power to detect a true difference of 2.18% in our primary outcome, at a two-sided type I error rate (alpha) of 0.05. Expecting a 10% dropout rate, we aimed to recruit a total of 66 participants.

The intention-to-treat (ITT) analysis population consisted of all participants who were randomised and received at least one dose of the assigned study intervention. The normality of continuous variables was tested using the Shapiro–Wilk and Kolmogorov–Smirnov tests. Within-group comparisons of study outcomes were made using a Wilcoxon signed-rank test. Between-group comparisons in changes in study outcomes were made using an ANCOVA linear model, with intervention group as fixed factor, and basal value of each outcome as covariate. Statistical analyses were performed in IBM SPSS statistics 23. For all analyses we used a significance level of 5% (0.05).

### Role of the funding source

The funding source of this study had no role in study design, data collection, data analysis, data interpretation or writing of the report. All authors had full access to the data in the study and had final responsibility for the decision to submit for publication.

## Results

**Figure 2:**
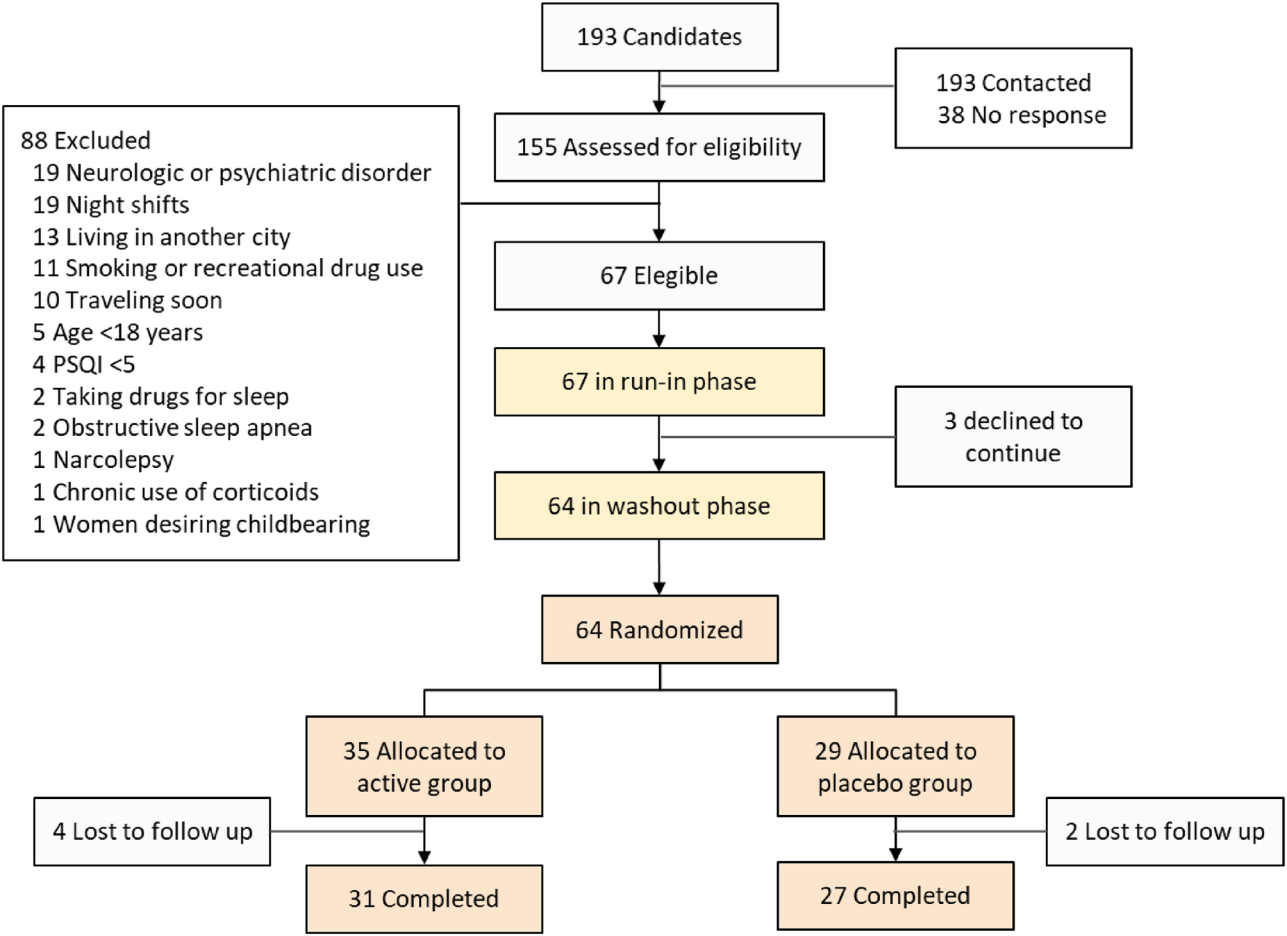
Study scheme.

Between January 16 and April 18, 2023, we screened 193 potential participants, 88 of whom were excluded due to neurologic or psychiatric disease, night shift work, smoking or recreational drug use, or other reasons (Fig. 2).

67 eligible individuals started the run-in phase, during which three patients decided not to go on with the study, while 64 continued to the washout phase. All 64 participants moved on to randomization, the minimization algorithm allocated 35 of them to the active group and 29 to the placebo group. The sex distribution, age, demographic, anthropometric, clinical and laboratory measures were similar between groups (Table 1). Mean basal PSQI and SF-36 scores and subscores were also comparable. Concerning actigraphy variables, all were similar except for sleep latency which was slightly longer in the placebo group. Throughout the 6 weeks of follow-up, there were in total four dropouts in the active group, and two in the placebo group (Fig. 2).

**Table 1:** Baseline characteristics of study participants.

	Intervention group				p-value
	Active		Placebo		
	n = 35		n = 29		
Women, n (%)	24	(68.6)	19	(65.5)	0.80
Age (years)	36.4	(14.4)	33	(13.0)	0.33
Educational level n (%)					
High school	3	(8.57)	1	(3.45)	0.79
College	5	(14.3)	7	(24.14)	
Technical/associate	2	(5.71)	1	(3.45)	
Professional	21	(60.0)	17	(58.6)	
Postgraduate	4	(11.4)	3	(10.3)	
Weight (kg)	63.9	(11.1)	67.2	(12.0)	0.26
Height (m)	1.63	(0.08)	1.64	(0.10)	0.79
Body-mass index (kg/m^2^)	23.9	(3.14)	24.9	(3.07)	0.20
Systolic blood pressure (mmHg)	108.2	(12.2)	111.2	(11.7)	0.31
Diastolic blood pressure (mmHg)	75.5	(6.99)	78.4	(7.02)	0.10
Heart rate	73.9	(11.3)	78.6	(8.85)	0.07
PSQI	9.60	(3.70)	9.86	(2.79)	0.75
Sleep quality	1.77	(0.60)	1.83	(0.54)	0.70
Sleep latency	2.34	(1.00)	2.41	(0.73)	0.75
Sleep duration	1.46	(0.92)	1.52	(0.78)	0.78
Sleep efficiency	1.17	(1.25)	1.14	(1.25)	0.91
Sleep disturbances	1.17	(0.57)	1.24	(0.44)	0.59
Daytime dysfunction	1.00	(0.77)	1.21	(0.90)	0.32
SF-36 score					
Physical functioning	95.4	(7.89)	92.9	(12.3)	0.33
Physical limitations	91.4	(23.4)	81.9	(31.3)	0.18
Emotional limitations	72.4	(39.2)	67.0	(42.7)	0.74
Energy	56.7	(18.6)	55.0	(18.9)	0.72
Emotional wellbeing	70.4	(16.9)	71.5	(14.8)	0.80
Social functioning	79.6	(18.5)	82.3	(19.6)	0.58
Pain	84.0	(16.7)	75.9	(20.9)	0.09
General health	69.3	(14.5)	64.5	(18.4)	0.25
Sleep latency (mean)	24.1	(26.4)	33.6	(31.3)	0.19
Total sleep time (min)	383.2	(55.4)	370.4	(50.8)	0.35
Sleep efficiency	0.84	(0.06)	0.81	(0.07)	0.052
Midsleep variability	1.03	(0.69)	1.00	(0.45)	0.83
Wake after sleep onset (minutes)	53.1	(12.9)	55.5	(14.9)	0.49
Salivary cortisol (nmol/L)	7.15	(3.99)	6.60	(4.27)	0.61
Blood glucose (mg/dL)	120.1	(70.3)	103.6	(27.1)	0.24
Urea nitrogen (mg/dL)	18.1	(6.33)	16.4	(5.28)	0.26
Creatinine (mg/dL)	0.91	(0.28)	0.85	(0.26)	0.38
Aspartate amino transferase (U/L)	14.4	(13.7)	12.6	(12.5)	0.57
Alanine amino transferase (U/L)	19.7	(9.21)	21.8	(8.15)	0.35
Bilirubin (mg/dL)	0.22	(0.20)	0.22	(0.12)	0.91
Thyroid-stimulating hormone (mUI/L)	2.58	(1.59)	2.67	(1.98)	0.85

Data are means (SD), unless indicated otherwise.

**Figure 3:**
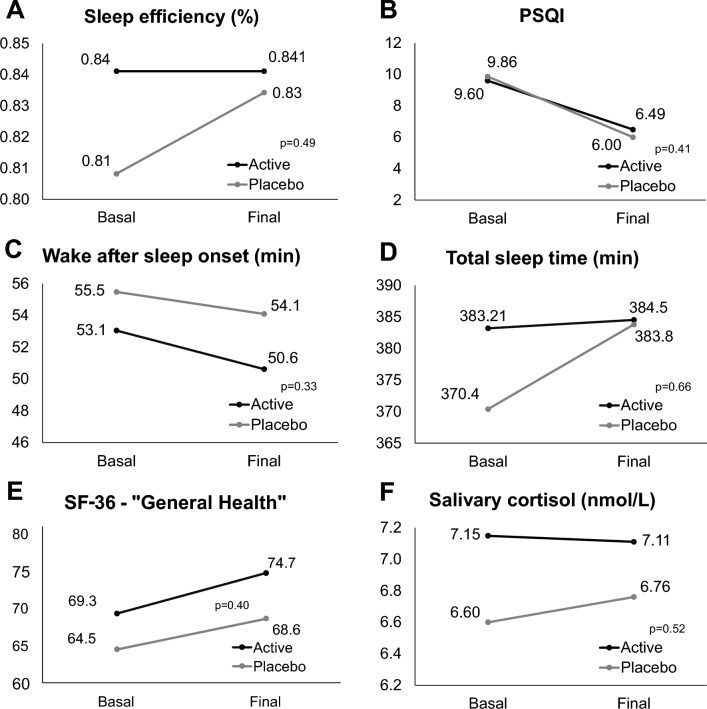
Change in primary and secondary outcomes, by intervention group. (**A**) Sleep efficiency. (**B**) PSQI score. (**C**) Wake-after sleep onset. (**D**) Total sleep time. (**E**) SF-36 subdomain 8: “General Health”. (**F**) Salivary cortisol. The p value represents the statistical significance of the between-groups difference in the final-basal change in each variable. *PSQI* Pittsburgh sleep quality index, *SF-36* 36-item health survey questionnaire.

The intention-to-treat analysis included 64 participants. In the within-group change analyses, sleep efficiency increased 3% in the placebo group (p = 0.049) but did not change in the active group (Table 2). The PSQI improved significantly in both groups (p < 0.001 in both cases), while the SF-36 score increased by 32.1 points with placebo (p = 0.032) and by 18.3 points with the active treatment (p = 0.077) (Table 2). Interestingly, both groups showed significant improvements in several PSQI domains (sleep quality, sleep latency, and sleep efficiency), except for sleep duration, which improved only in the placebo group. In subdomain 8 of the SF-36 (“General Health”), both groups experienced a significant increase, of 5.43 points in the active group, and of 4.14 points in the placebo group. Nonetheless, the between-group difference was not significant (Fig. 3E).

**Table 2:** Within-group change in study outcomes.

	Active		Placebo	
	Final-basal	p-value	Final-basal	p-value
Change in sleep efficiency (%)	0.00	0.71	0.03	0.05
Change in PSQI	−3.11	< 0.001	−3.86	< 0.001
Change in sleep quality	−0.66	< 0.001	−4.15	0.00
Change in sleep latency	−0.86	< 0.001	−0.69	< 0.001
Change in sleep duration	−0.23	0.11	−1.17	0.02
Change in sleep efficiency	−0.49	0.04	−0.41	0.02
Change in sleep disturbances	−0.11	0.21	−0.62	0.29
Change in daytime dysfunction	−0.17	0.31	−0.52	0.14
Change in WASO (minutes)	−2.43	0.20	−0.31	0.83
Change in TST (minutes)	1.33	0.91	13.00	0.28
Change in SF-36	18.30	0.08	32.10	0.03
Change in physical functioning	−0.14	0.97	0.17	0.70
Change in physical limitations	2.86	0.19	7.76	0.04
Change in emotional limitations	−0.01	0.95	8.03	0.38
Change in energy	4.71	0.07	3.62	0.13
Change in emotional wellbeing	2.29	0.13	1.10	0.99
Change in social functioning	1.79	0.19	3.45	0.54
Change in pain	1.36	0.36	4.57	0.07
Change in general health	5.43	0.01	4.14	0.02
Change in salivary cortisol (nmol/L)	0.03	0.71	−0.40	0.95

p-values are within-group, from a Wilcoxon test.

For our primary outcome, sleep efficiency, the between-group difference in the change was not significant (p = 0.49) (Fig. 3A). Both groups experienced a noticeable improvement in the PSQI, a decrease of 3.86 points (39.1%) in the placebo group, and of 3.11 points (32.3%) in the active group (between-groups p-value 0.41) (Fig. 3B). In actigraphy variables, the mean WASO also decreased in both groups, by 2.4% in the placebo arm, and by 4.6% in the active treatment arm (Fig. 3C). Total sleep time increased more in the placebo (13.0 min) than in the active group (1.33 min), but the difference was not significant (p = 0.66) (Fig. 3D). There were improvements in quality of life in both groups, with the mean SF-36 score increasing by almost twice as much in the placebo group relative to the active treatment. Nonetheless, the between-group difference was not significant (p = 0.68) (Fig. 3E). Salivary cortisol remained relatively stable in both groups (− 0.40 nmol/L ion placebo, + 0.03 nmol/L in active, p = 0.52) (Fig. 3F).

There were no serious adverse events during the trial. The frequency of reported adverse events is displayed in Table 3. The most frequently reported adverse events were gastrointestinal in both groups: 22.9% of the participants in the active and 10.3% in the placebo group referred heartburn, while bloating was reported by 14.3% and 3.4%, respectively. Daytime sleepiness was more frequent in the active (17.1%) than in the placebo group (10.3%). One participant from the placebo group reported a poorer quality of sleep. All adverse events improved after the first week of intervention and had resolved by the end of the follow-up period. At the end of week 6, the mean adherence rate was 80.9% in the active group and 79.4% in the placebo group.

**Table 3:** Frequency of adverse events.

Adverse events	Group	
	Active	Placebo
	n = 35	n = 29
Nausea	1 (2.9%)	2 (6.9%)
Diarrhea	0 (0%)	2 (6.9%)
Constipation	1 (2.9%)	1 (3.4%)
Heartburn	8 (22.3%)	3 (10.3%)
Palpitations	1 (2.9%)	0 (0%)
Daytime sleepiness	6 (17.1%)	3 (10.3%)
Skin lesions	0 (0%)	0 (0%)
Hypoprosexia	0 (0%)	1 (3.4%)
Dysgeusia	3 (8.6%)	1 (3.4%)
Others	10 (28.6%)	4 (13.8%)
Bloated stomach	5 (14.3%)	1 (3.4%)
Abdominal pain	2 (5.7%)	0 (0%)
Early awakening	1 (2.9%)	0 (0%)
Nightmares	1 (2.9%)	0 (0%)
Low libido	1 (2.9%)	0 (0%)
Headache	0 (0%)	1 (3.4%)
Night sweats	0 (0%)	1 (3.4%)
Poorer sleep	0 (0%)	1 (3.4%)

Data are n (%).

Laboratory parameters showed no indication of renal or hepatic impairment in either group (Table 4).

**Table 4:** Changes in laboratory parameters, by intervention group.

	Group				Between-groups p-value
	Active		Placebo		
Blood glucose (mg/dL)	−10.7	31.7	−7.14	31.6	0.66
Urea nitrogen (mg/dL)	−0.42	7.09	−1.97	7.60	0.40
Creatinine (mg/dL)	0.09	0.28	0.10	0.27	0.85
AST (U/L)	−3.33	16.1	−4.18	11.2	0.81
ALT (U/L)	−8.57	12.5	−13.2	10.6	0.12
Bilirubin (mg/dL)	0.13	0.29	0.17	0.27	0.61

Data are means (SD).

## Discussion

Nutraceutical products have been proposed as a treatment alternative for sleep disturbances. This study assessed the effect of a nutraceutical combination on objective and subjective sleep parameters during 6 weeks of intervention among adults with impaired sleep. Our primary outcome, sleep efficiency, remained unchanged in the intervention group and improved in the placebo group, but the difference between groups was not statistically significant. For patient-reported outcomes like sleep quality (measured by PSQI) and health-related quality of life (measured by SF-36), there were improvements regardless of intervention group. On the other hand, other objective variables measured by actigraphy showed a slightly different result according to the intervention. In the active group the reduction in WASO was larger than in the placebo group, while total sleep time improved markedly in the placebo group. Salivary cortisol levels remained relatively stable in both groups.

In concordance with our results, a prior clinical trial of valerian extract among older women with insomnia found no significant effect on sleep efficiency, but a trend towards a benefit in WASO with the supplementary intervention^31^. Likewise, a randomised study of saffron against placebo in adults with mild to moderate sleep disorders and anxiety, found no between-groups difference in the change in sleep efficiency^32^. Concerning other components of the study intervention, there was a host of prior evidence suggesting a positive impact of lemon balm and green tea on sleep quality, but these studies had derived sleep efficiency from PSQI component 4, rather than directly measuring it^33,34^. Interestingly, the small 3% increase in sleep efficiency in the placebo group reached within-group significance. This finding highlights the potential relevance of the placebo effect even on objective sleep measurements^35,36^.

One of the outcomes most related to sleep quality is WASO, the amount of time spent in the wake state after initially having fallen asleep. In our study, WASO decreased in both groups, but the change was not significant. Moreover, total sleep time increased in the placebo group by 13 min, but this change was also not statistically significant. A clinical trial in healthy young woman assessed the acute effect of green tea extract on sleep and polysomnographic variables, registering no difference in WASO and total sleep time between green tea extract and placebo^37^.

PSQI summarizes in a single score the perceived quality of sleep and represents a true measure of the burden imposed by sleep disturbances. Participants in the active and placebo group started with a comparable PSQI, after 6 weeks of intervention both groups had a notable 3 point-improvement in their mean total score. A study of green tea extract among healthy Japanese adults found a substantial improvement in PSQI with the intervention, but like in our study, it was not significantly different from placebo^34^. Moreover, when the presumed active component of tea extract (L-theanine) was combined with magnesium, B-vitamins and Rhodiola in a single nutraceutical supplement, its effect on the PSQI among adults who scored high on a stress questionnaire was comparable to placebo^38^. Despite initially encouraging results among post-menopausal women^33^, the evidence on the efficacy of lemon balm for sleep quality has yielded heterogeneous results. Haybar H et al., assessed the effect of lemon balm or placebo on the PSQI among patients with chronic stable angina, both groups had equally significant reductions in the PSQI^39^. Contrastingly, a study in patients with type 2 diabetes plus depression or anxiety symptoms found no significant modification in the PSQI after 4 weeks of lemon balm supplementation^40^. In patients with insomnia, a 4 week study of a combination of lemon balm with the plant *Nepeta menthoides* evidenced a PSQI improvement significantly different from placebo^41^. A host of prior evidence suggested an effect of valerian on sleep quality. A meta-analysis of six studies comparing valerian mono-preparations to placebo indicated statistically significant placebo-subtracted reductions in the PSQI^42^, albeit with a large between-trial heterogeneity (I^2^ = 93%). Unfortunately, such results were not replicated in our study.

We observed a very large improvement in the PSQI, regardless of intervention group. The magnitude of the placebo effect can be unusually high for sleep-related outcomes: a meta-analysis of 82 treatment groups from 32 clinical trials reported a mean placebo effect of 61% on subjective sleep parameters^35^. Hence, the inclusion of a placebo control is of utmost importance in studies that assess the efficacy of any intervention for sleep, nutraceuticals included. In parallel to the improvements in PSQI, participants from both groups increased their SF-36 scores. This is an anticipated result, as quality of sleep greatly influences health-related quality of life ^43,44^.

Multiple factors can explain the results of our study, in which the intervention and placebo group experienced similar improvements in key sleep outcomes. Many effective interventions for sleep disturbances are behavioural in nature, including relaxation training, stimulus control therapy, sleep restriction therapy, sleep hygiene, paradoxical intention therapy, cognitive restructuring, and many others^45^. In our study, participants had to follow a pre-bed routine that included tasks like warming and drinking a beverage, which may have induced a particular disposition, more favourable to sleep. This implicit behavioural and cognitive influence may partially explain the positive effect in both groups, without evidence of an added benefit from the composition of the tested intervention. Some of the favourable changes may also reflect a plausible Hawthorne effect from feeling observed while wearing the actigraphy device^46^.

The proportion of adverse events was low and very similar between groups, and we found no evidence of renal or hepatic impairment.

Strengths of our study include its randomised design, careful collection and control of study variables, provision of the study intervention and close monitoring of adherence. Also, the combination of objective and subjective outcome measures of sleep quality provided a more holistic assessment of sleep. We assessed not only sleep itself, but variables closely related to it and with clinical or biological relevance, such as health-related quality of life or salivary cortisol, information that is not always collected in studies of non-pharmacological interventions for sleep. Concerning the intervention, the simultaneous assessment of various nutraceutical products with prior indications of positive effects on sleep, increased the probability of evidencing a beneficial effect, if it was present. The central limitations of our study are its duration of only 6 weeks, although most evidence shows that sleep-related variables can be substantially modified in such timespan^47^, and its relatively modest sample size. Also, we performed thyroid function tests only before inclusion in the trial. Another phenomenon that we cannot rule out is the existence of antagonism among the components of the nutraceutical intervention, so that one component may partially antagonize the effects of another, we had no mechanism for testing this hypothesis. Moreover, it is also possible that participants communicated among them leading to some degree of unmasking, but there was no way for the participants to tell the particular organoleptic characteristics of each intervention. Lastly, we could not rule out that some participants might have sleep disorders without a formal diagnosis or treatment. However, any potential influence from such individuals would exist in both groups due to randomization including minimization by a sleep-quality parameter (PSQI). Importantly, our results pertain to the general population with impaired sleep, not necessarily to individuals with a formal diagnosis of insomnia.

Future research efforts should focus on the use of nutraceuticals as part of a broader behavioural strategy aimed at patients with sleep disturbances. In this context, the inclusion of an appropriate comparator will be of extreme relevance. Larger studies of the efficacy and safety of nutraceuticals (including excessive daytime sleepiness) are needed, in order to inform evidence-based recommendations about products from this growing market.

In conclusion, in this clinical trial among adults with impaired sleep, a nutraceutical combination did not improve sleep compared to a placebo infusion. This result may be explained by the influence of behavioural and cognitive factors.

## Data Availability

De-identified data and a data dictionary will be available upon request to the corresponding author according to a formal research proposal. The data will be available only for research and non-commercial purposes carried out by individuals with academic or public health institutions. Data access will need agreement and approval by the local ethics committee.
